# Study of weight and height development in children after adenotonsillectomy

**DOI:** 10.1016/S1808-8694(15)30573-5

**Published:** 2015-10-19

**Authors:** Alexandre Augusto Fernandes, Thiago Alves Alcântara, Daniel Vasconcelos D’Ávila, Jeferson Sampaio D’Ávila

**Affiliations:** 1 M.Sc in Health Sciences - UFS/SE, Otorhinolaryngology Physician.; 2 Medical Student.; 3 Medical Student.; 4 MD. ENT - School of Medicine of São Paulo University, Otorhinolaryngology Professor at Sergipe Federal University.

**Keywords:** adenotonsillectomy, child, anthropometrical measurements, growth delay

## Abstract

The daily clinical observation of weight-height growth delays in children with obstructive hypertrophy of the pharyngeal and palatine tonsils is a workaday practice in pediatric otorhinolaryngology, and the surgical correction of this condition, when properly done in time, through adenotonsillectomy, can lead to a “catch up growth”.

**Aim:**

To investigate the real weight-height gain present in this population when they are surgically treated.

**Materials and Methods:**

Through a clinical prospective study, two groups of children carrying pharyngopalatine hypertrophy were followed up: group 1 was submitted to surgical intervention, and group 2 was not. All patients underwent standardization of anthropometrical measurements (weight and height), including their age-related percentiles, in the beginning and at the end of 06 (six) months.

**Results:**

While group 1 increased its height average in relation to the initial average in 6.66cm, the control group increased its average in 1.9cm (p=0.0004). In relation to weight, group 1 increased 2150g in average, while group 2 presented an average increase of 690g (p=0.0010).

**Conclusions:**

The children that underwent adenotonsillectomy acquired a higher weight-height growth potential in relation to those children who were not operated.

## INTRODUCTION

Hyperplasic and hypertrophic processes of pharyngeal and palatine tonsils are very prevalent events in the general population, mainly the pediatric one, compromising the respiratory pattern through upper airway obstruction.

Adenotonsillar hyperplasia shows a clinical picture with a number of general signs and symptoms. Among them: obstructive sleep apnea, weight and height gain disorders, mouth breathing and its craniofacial repercussions, chewing and swallowing problems, increase of upper airway infections and lung alterations such as cor pulmonale and pulmonary hypertension^1^, ^2^, ^3^, ^4^.

It is well known that children with obstructive tonsillar hyperplasia have a worsening in their quality of life. This is mainly due to obstructive sleep apnea which, for some authors, is the main morbidity associated^5^. A clinical daily observation, with scientific confirmation, allows us to establish a direct relationship between patients with adenotonsillar hyperplasia with the onset of weight and height gain deficit that gets worse when associated with obstructive sleep apnea and hypoapnea (OSAH). Palatine tonsillar hyperplasia corresponds to approximately 75% of OSAH in children, and within this group 1% to 46% may show growth delays^6^.

OSAH causes sleep disorders resulting in the alteration of growth hormone (GH) physiological secretion, which happens in a pulsating manner, mainly at night, having a very close relationship with the experienced sleep pattern^7^. Thus, patients with obstructive palatine tonsillar hyperplasia with OSAH may develop growth deficit from the alteration in the GH-IGF-1 axis^8^.

## GOAL

To evaluate weight and height gain evolution in patients with obstructive adenotonsillar hyperplasia who underwent adenotonsillectomy.

## METHOD

After the evaluation and authorization by the Human Research Ethics Committee (protocol nº085/04 CEP/UFS), we included in this study 22 patients with pharyngeal and palatine tonsil hypertrophy clinically diagnosed with obstructive sleep apnea. All these patients belong to the pediatric age range, they belonged to the same social class and they were seen at the same otorhinolaryngology public service. Patients were divided into two groups. Group 1 (those who underwent surgery) with 12 patients and group 2 with 10 patients (control group) who did not have any surgery done.

Group 1 age (case) varied from 03 years and 04 months of age to 09 years and 08 months of age with an average of 06 years and 01 month. Group 2 (control group) with ages varying between 04 years of age to 10 years and 03 months of age, with an average of 06 years and 10 months of age. Regarding gender, group 1 (those who underwent surgery) had 07 male patients and 05 female patients, and group 2 (control group) had 05 male and 05 female patients.

Everyone (cases and control patients) had a detailed anamnesis, a general and specialized physical examination and filled in an otorhinolaryngological clinical evaluation form. Among the inclusion criteria, there was videonasopharyngoscopic study with pharyngeal tonsil diagnosis grade III2 and palatine tonsils with Brodsky^9^ grades +2, +3 and +4 through oropharynx examination. Patients with other upper airways obstructive pathologies were excluded from this study, as well as patients with some type of counter indication for surgical treatment.

An anthropometric evaluation form was filled in checking weight and height and their percentages according to age and gender through the growth curve graph developed by the National Center for Health Statistics.

Children selected for group 1 were the first 12 who would undergo surgery in the months of July and August of 2005 and those 10 children who belonged to the control group were seen in August 2005, and due to the high demand, they were scheduled for surgery on the first semester of 2006.

## RESULTS

### Height

First we assessed the initial heights of both groups. The average initial heights of groups 1 and 2 were 114 cm and 120.1cm each. It was seen that this initial difference was not statistically significant (p=0.3771), a fact that pointed towards the homogeneity among the groups regarding the variable height.

The initial homogeneous distribution was confirmed by the percentage analysis. For assessing percentages, through an analogy of values we changed a categorical variable, such as the case of height-age and weight –age percentages into an ordinal variable, as it was the case of the numerical sequence of 1 (one) to 13 (thirteen). Thus, the percentages (p) obtained through the growth curve graphs, both for height by age and for weight by age: p5, p5-10, p10, p10-25, p25, p25-50, p50, p50-75, p75, p75-90, p90, p90-95 and p95 were made into percentage value 1, percentage value 2, percentage value 3 and so forth until percentage value ^13^.

Analyzing the initial distribution of percentages for height, we obtained 6.58 and 6.9 of average for height percentage values in groups 1 (those patients who underwent surgery) and group 2 (control group) each, which did not show any statistical difference (p=0.3115).

After 6 months of surgery, the group of patients who had surgery showed a final height average of 121.08cm. The control group reached the end of the study with a final height average of 122cm. (Tables 1 and 2).

**Table 1:** Average of initial and final heights (cm) in group 1 (who underwent surgery) and t-Student test.

Group 1 initial average	Group 1 final average	Standard deviation of initial Group 1	Standard deviation of final Group 1	t	p
114,4	121,1	15,6	14,0	-7,2160	0,0000

**Table 2:** Average of initial and final heights (cm) in group 2 (control) and t-Student test.

Initial average in Group 2	Final average in Group 2	Standard deviation of initial Group 2	Standard deviation of final Group 2	t	p
120,1	122,0	13,4	14,2	-3,77	0,0044

Although we observed that both groups grew, the differences between the averages of final and initial heights of the group of patients who underwent surgery was greater than those of the control group. While the group who had surgery increased their final height average regarding the initial average in 6.66cm, the control group increased its height average in only 1.9cm. (Table 3)

**Table 3:** Average of differences between final and initial heights in groups 1 (with surgery) and group 2 (control) and t-Student test.

Average of Group 1	Average of Group 2	Standard Deviation of Group 1	Standard Deviation of Group 2	t	P
6,7	1,9	3,2	1,6	4,28	0,0004

Through the final percentage evaluation for height – age, it was confirmed that in the group of patients who had surgery, the percentage had evolved for a median value of p75, or percentage value 9, whereas the control group showed reduction of the median value for p25-50, that is to say, percentage value 6. With the application of the statistical test, the final average found for percentage values was 8.75 for the group of patients who had surgery and 6.6 for the control group. (Table 4)

**Table 4:** Average of final percentage value for height in groups 1 (surgery) and 2 (control) and t-Student test.

Average of Group 1	Average of Group 2	Standard Deviation of Group 1	Standard Deviation of Group 2	t	p-value
8,75	6,60	2,22	1,65	2,53	0,0198

### Weight

Homogeneity of groups was confirmed in relation to the initial weight from the moment that any statistical difference was found among the averages of initial weights of those patients who had surgery and those of the control group (p = 0.8449). The group who had surgery had an initial weight average of 24,100g whereas the control group had an average of 23,630g.

In addition, statistical match was found among groups form the average of percentage values (weight-age), with 8.58 for those patients who had surgery and 6.5 for the control (p = 0.1408).

The comparison of initial and final weight averages of the control revealed a progressive weight evolution. Group 1 increased its weight 2,150g on average, reaching 26,250g and group 2 in 690g reaching 24,320g. Weight gain among groups, as well as differences among initial and final averages for weight of groups 1 and 2 showed statistic significance. (Tables 5, 6 and 7)

**Table 5:** Average of initial and final weight (g) of group 1 (with surgery) and t-Student test.

Initial average of Group 1	Final average of Group 1	Standard deviation of initial Group 1	Standard deviation of final Group 2	t	P
24.100	26.250	8.107	7.677	-7,47	0,0000

**Table 6:** Average of initial and final weight (g) of group 2 (control) and t-Student test.

Initial average of Group 2	Final average of Group 2	Standard deviation of initial Group 2	Standard deviation of final Group 2	t	p
23.630	24.320	8.579	8.822	-3,00	0,0150

**Table 7:** Average of differences between final and initial weight of groups 1 (with surgery) and 2 (control) and t-Student test.

Average of Group 1	Average of Group 2	Standard deviation of Group 1	Standard deviation of Group 2	t	p
2.150	690	997	728	3,85	0,0010

The average of final percentage values was 10.08 for group 1 and 6.0 for group 2. This final analysis shows an increasing positive change of weight percentage for those patients who had surgery in relation to patients in the control group. (Table 8)

**Table 8:** Average of final percentage value for weight in groups 1 (with surgery) and 2 (control) and t-Student test.

Average in Group 1	Average in Group 2	Standard deviation of Group 1	Standard deviation of Group 2	t	p-valor
10,08	6,00	2,23	3,37	3,40	0,0028

## DISCUSSION

Very commonly and especially in the pediatric age range, palatine tonsils suffer hyperplasic processes due to the increase of cells and/or hypertrophy with the increase in cell size, promoting an increase of the organ volume, contributing to a series of signs and symptoms which are interesting for the otorhinolaryngology practice and its related specialties.

Among the clinical signs mostly seen in children with adenotonsillar hyperplasia, there is growth and development delay. This concern is due to the fact that this is a daily work in otorhinolaryngology and pediatric practices that may involve issues of psychosomatic and socio-economic nature, besides the lack of a conclusive and agreed explanation in the specialized literature.

There is also the fact that through a properly indicated surgical treatment at the right time, this procedure may resume growth, the so called after surgical stature “stretch”, which even makes possible a change in height and weight percentages^10^. From this evidence, despite still controversial for some given situations, literature already indicates growth deficit as one of the criteria for absolute indication of adenotonsillectomy^11^.

With homogenous groups among themselves (group 1 and 2) and using for the first time a control group, this investigation shows the benefits of adenotonsillectomy. The statistical importance (p<0.05) reached by the weight and height gain development in patients who had adenotonsillectomy in comparison to the control group, using the measurements of absolute and percentage values that increase the accuracy of the evaluation through the use of other variables such as age and gender, indicates surgical treatment as the most appropriate treatment modality.

## CONCLUSION

For children with obstructive adenotonsillar hyperplasia, mainly with evidence of growth delay, adenotonsillectomy is absolutely indicated. This surgery promotes weight and height gain specially in those patients with sings that suggest sleep apnea.
